# Associations among quality of life, depressive symptoms, body shape concerns, anthropometric characteristics, and diet quality in women with PMOS: a cross-sectional study

**DOI:** 10.3389/fnut.2026.1928327

**Published:** 2026-10-13

**Authors:** Esra Irmak, Nevin Sanlier

**Affiliations:** Department of Nutrition and Dietetics, Faculty of Health Sciences, Ankara Medipol University, Ankara, Türkiye

**Keywords:** body shape, depression, diet quality, PMOS, quality of life

## Abstract

**Background:**

Polyendocrine metabolic ovarian syndrome (PMOS) is a prevalent endocrine disorder associated with impaired quality of life, body image dissatisfaction, and psychological distress. This study aimed to evaluate the associations between quality of life, depression, body shape concern, and diet quality among women with PMOS.

**Methods:**

This cross-sectional study was conducted between July and December 2022 at a training and research hospital in Ankara, Türkiye, and included 247 women aged 19–40 years diagnosed with PMOS according to the Rotterdam criteria (2003). Participants were assessed using anthropometric measurements, Polycystic Ovary Syndrome Questionnaire (PCOSQ-50), Body Shape Questionnaire (BSQ-34), Beck Depression Inventory (BDI), Healthy Eating Index (HEI-2010), Carbohydrate Quality Index (CQI), and Dietary Inflammatory Index (DII). Group comparisons, correlation analyses, and multiple linear regression analyses were performed to examine associations among the study variables.

**Results:**

Participants with minimal depressive symptoms reported significantly higher quality of life scores compared to those with mild or moderate-to-severe depression (*p* < 0.001). Higher BMI was associated with greater body shape concern (*p* < 0.001), and greater body shape concern was associated with lower PCOSQ-50 scores (*p* < 0.001). Significant negative correlations were observed between PCOSQ-50 scores and BSQ-34, BDI, and BMI (*p* < 0.05).

**Conclusion:**

These findings demonstrate significant associations among quality of life, depressive symptoms, body shape concerns, and BMI in women with PMOS and underscore the importance of considering psychosocial and anthropometric factors alongside dietary characteristics in the comprehensive assessment of women with PMOS.

## 1 Introduction

Polyendocrine metabolic ovarian syndrome (PMOS) is a prevalent and heterogeneous endocrine-metabolic disorder characterized by reproductive, metabolic, endocrine, dermatological, and psychological manifestations (1, 2). PMOS affects approximately one in eight women worldwide, although the prevalence of the condition varies according to the diagnostic criteria and characteristics of the population studied (2–4). Beyond its reproductive and metabolic manifestations, PMOS is increasingly recognized as a condition associated with a substantial psychological and quality-of-life burden (5). The clinical manifestations of PMOS, including menstrual irregularities, infertility, excess weight, hirsutism, and acne, have the potential to impact a wide range of domains, including emotional wellbeing, self-perception, social functioning, and daily life. This emphasizes the multidimensional impact of PMOS on affected women (1, 5).

Psychological and body image-related concerns represent significant components of this burden. The prevalence of depressive and anxiety symptoms is increased in women diagnosed with PMOS. These psychological features have the potential to substantially impair health-related quality of life (5). Furthermore, PMOS-related appearance-affecting symptoms, including obesity, hirsutism, and acne, have been demonstrated to have a detrimental effect on body image and body satisfaction (6, 7). Recent meta-analytic evidence indicates that women diagnosed with PMOS experience elevated levels of body image concerns in comparison with women not diagnosed with PMOS, particularly with regard to appearance, weight, and satisfaction with specific body areas (7). It is important to note that these relationships should not be interpreted as evidence that negative body image directly causes eating disorders or psychological distress. Instead, the presence of body dissatisfaction, psychological symptoms and disturbed eating behaviors may coexist and interact within the complex psychosocial burden of PMOS (5, 7). Eating disorders and disordered eating are also more prevalent among women with PMOS, with recent evidence demonstrating higher odds of eating disorders, particularly binge-eating disorder and bulimia nervosa, compared with women without PMOS (8, 9).

It is hypothesized that dietary factors may represent another important dimension associated with the clinical and psychosocial characteristics of PMOS. While healthy lifestyle behaviors, encompassing a nutritious diet and regular physical exertion, continue to be pivotal components of PMOS management, contemporary evidence underscores health benefits that extend beyond mere weight management (5, 10). Furthermore, contemporary international recommendations do not advocate a singular dietary composition as optimal for all women with PMOS, instead emphasizing sustainable, individualized healthy eating patterns (5). This broader perspective is of particular relevance given that dietary characteristics have the potential to be associated not only with metabolic health but also with psychological wellbeing. Recent evidence suggests that dietary factors and overall diet quality may be associated with depressive symptoms among women with PMOS (11).

Beyond the overarching concept of overall healthy eating, specific dimensions of dietary quality may offer supplementary insights into the comprehensive health burden of PMOS. The quality of carbohydrates may be of particular relevance, as emerging evidence suggests that the sources and quality of dietary carbohydrates, including whole-grain, refined-grain, and fiber intake, may be more informative than carbohydrate quantity alone in women with PMOS (12). The inflammatory potential of the diet may also be important because chronic low-grade inflammation is implicated in the pathophysiology of PMOS (1, 13). Recent studies evaluating the Dietary Inflammatory Index (DII) have further suggested associations between a diet that is more pro-inflammatory and inflammatory and cardiometabolic characteristics in women with PMOS (14, 15). The findings, when considered collectively, provide a rationale for evaluating overall healthy eating, carbohydrate quality, and dietary inflammatory potential in relation to the broader clinical and psychosocial features of PMOS.

Despite the growing emphasis on dietary factors in PMOS, previous research has primarily concentrated on metabolic, anthropometric, and reproductive outcomes. The relationships between different dimensions of diet quality and patient-centered outcomes remain less well characterized. In particular, there is a paucity of evidence that has simultaneously evaluated overall healthy eating, carbohydrate quality, and dietary inflammatory potential in relation to quality of life, body shape, and depressive symptoms among women with PMOS. Addressing this knowledge gap may provide a more integrated understanding of how dietary characteristics relate to the multidimensional burden of PMOS. The present study therefore sought to assess the relationships of healthy eating, carbohydrate quality, and dietary inflammatory potential with quality of life, body shape, and depression among 247 women with PMOS aged 19–40 years.

## 2 Materials and method

### 2.1 Participants

This study was conducted between July and December 2022 in a training and research hospital in Ankara and included 247 women aged 19–40 years who presented to the outpatient clinic, were diagnosed with PMOS according to the Rotterdam criteria (2003), and voluntarily agreed to participate in the study. The inclusion criteria were being aged 19–40 years, having a diagnosis of PMOS according to the Rotterdam criteria, and volunteering to participate in the study. The exclusion criteria were pregnancy or breastfeeding and the presence of a diagnosed psychiatric disorder requiring current pharmacological treatment. Ethical approval was obtained from the University Health Sciences Non-Invasive Research Ethics Committee (Decision No. 137, 18.07.2022), and informed consent was obtained in accordance with ethical standards for human research.

### 2.2 Anthropometric measurements

Participants' waist, hip, neck and mid-upper arm circumferences were measured using a non-stretchable measuring tape according to standardized procedures (16). Body fat percentage (%) and lean body mass (kg) were estimated using a TANITA BC-601 body-composition analyzer based on bioelectrical impedance analysis (BIA).

### 2.3 Instruments

The Polycystic Ovary Syndrome Questionnaire (PCOSQ-50) is a 50-item instrument designed to assess the specific quality of life of individuals diagnosed with PCOS over the previous 4 weeks. The instrument comprises six domains: psychosocial and emotional status (12 items), fertility (7 items), sexual function (9 items), obesity and menstrual disorders (9 items), hirsutism (6 items), and coping (7 items). The items are scored on a 5-point Likert scale, with “always” assigned a score of 1, “often” assigned a score of 2, “sometimes” assigned a score of 3, ‘rarely' assigned a score of 4 and ‘never' assigned a score of 5. The PCOSQ-50 total score was calculated by summing the item scores, yielding a possible total score ranging from 50 to 250, with higher scores indicating better PMOS-specific quality of life. Domain scores were calculated by summing the item scores within each respective domain and were used to evaluate specific dimensions of PMOS-related quality of life. The Cronbach's alpha value of the scale subscales in the validation study was 0.88, 0.85, 0.85, 0.81, 0.83, and 0.61, respectively, and the total Cronbach's alpha value was 0.88 (17). In the present study, Cronbach's alpha values for the six domains were 0.87, 0.88, 0.97, 0.78, 0.98, and 0.75, respectively, and 0.91 for the total scale. The total score was considered the overall measure of PMOS-specific quality of life, whereas domain scores were examined to characterize specific dimensions of PMOS-specific quality of life.

The Body Shape Questionnaire (BSQ-34) is a 34-item unidimensional instrument designed to assess concerns about body shape. The items are scored on a 6-point Likert scale ranging from 1 (never) to 6 (always), yielding a total score ranging from 34 to 204, with higher scores indicating greater body shape concern. Scores falling below 80 indicate no body shape concern, 81–110 indicate mild concern, 111–140 indicate moderate concern, and scores above 140 indicate severe concern. While the Cronbach's alpha value of the scale was 0.97 (18), Cronbach's alpha was 0.92 in the present study.

The Beck Depression Inventory (BDI) is a 21-item instrument utilized for the assessment of depressive symptoms in adults (19). Each item in the scale consists of four response options, scored from 0 to 3, yielding a total score ranging from 0 to 63, with higher scores indicating greater severity of depressive symptoms. The severity of depression is categorized into four distinct classifications: minimal (0-9), mild (10-16), moderate (17-29), and severe (30-63). The Cronbach's alpha value of the scale is 0.80 (19). In the present study, Cronbach's alpha was 0.82.

### 2.4 Nutritional status assessment

The subject's dietary intake was assessed by means of a 24-h dietary recall. In order to enhance their capacity for estimating portion sizes, the participants were presented with standardized images of foods that are commonly consumed in Türkiye (20). Nutrient and energy intakes were calculated using the BeBiS nutrition software (21). The quality of the diet was evaluated using three complementary indices: the Healthy Eating Index-2010 (HEI-2010), Carbohydrate Quality Index (CQI), and Dietary Inflammatory Index (DII) (22–24).

The HEI-2010 is a tool designed to evaluate the overall quality of an individual's diet and the degree to which they adhere to recommended dietary guidelines. The tool employs a scale that ranges from 0 to 100, with higher scores denoting better diet quality. Scores of 80 and above were classified as “good,” 51–80 as “fair,” and ≤ 50 as “poor” (22). The CQI evaluates carbohydrate quality based on four components: glycemic index, dietary fiber intake, the ratio of whole-grain to total-grain carbohydrates, and the ratio of solid carbohydrates to total solid and liquid carbohydrates. Each component is assigned a score ranging from 1 to 5, with the glycemic index reverse-scored, thereby yielding a total score ranging from 4 to 20. Higher scores thus indicate better carbohydrate quality (23). The DII is a tool used to assess the inflammatory potential of a diet. The original index was first introduced by Cavicchia et al. (25) and was subsequently expanded to 45 dietary parameters by Shivappa et al. (24). Higher DII scores are indicative of a diet that is more pro-inflammatory, whereas lower scores are indicative of a diet that is more anti-inflammatory (24).

### 2.5 Statistical analysis

SPSS (Statistical Package for Social Sciences) statistical package program version 22 was used for statistical evaluation of the data. Evaluation of categorical variables was made with the Chi-Square (χ^2^) test. The distribution of continuous variables was evaluated before parametric analyses. Independent-samples *t*-tests were used for comparisons between two independent groups, whereas one-way analysis of variance (ANOVA) was used for comparisons among three or more independent groups. When the overall ANOVA was significant, Tukey's *post-hoc* test was used for pairwise comparisons. Relationships between continuous variables were evaluated using Pearson's correlation coefficient (*r*).

Multiple linear regression analyses were performed separately with PCOSQ-50 total score, BSQ-34 score, and BDI score as dependent variables. Age group, marital status, exercise status, comorbidity, BMI, duration since PMOS diagnosis, CQI, DII, and HEI-2010 scores were included as independent variables in each model. For categorical predictors, the reference categories were 19–24 years for age group, single for marital status, non-exercise for exercise status, and absence of comorbidity for comorbidity status. Assumptions of multiple linear regression were evaluated before interpretation of the models. Linearity and homoscedasticity were assessed by visual inspection of standardized residuals plotted against standardized predicted values. Residual distribution was evaluated using histograms and normal P–P plots. Potential influential observations were assessed using standardized residuals, leverage values, and Cook's distance. Standardized residuals outside ± 3 were considered indicative of potential outlying observations, and Cook's distance values >1 were considered indicative of potentially influential observations. Multicollinearity was evaluated using tolerance and variance inflation factor (VIF) values; tolerance values >0.20 and VIF values < 5 were considered acceptable. Residual independence was additionally assessed using the Durbin–Watson statistic. Regression results were reported as unstandardized coefficients (B), standard errors (SE), standardized coefficients (β), 95% confidence intervals (CI), *p*-values, VIF, tolerance and adjusted R^2^ values.

There were no missing data for the variables included in the statistical analyses.

No correction for multiple testing was applied across analyses involving the PCOSQ-50 total and domain scores.

A two-sided *p* < 0.05 was considered statistically significant.

## 3 Results

The mean age of the participants was 25.9 years (±5.0 years). Of the 247 participants, 116 (47.0%) were aged 19–24 years and 131 (53.0%) were aged 25–40 years. With respect to educational attainment, 40 (16.2%) had completed primary or secondary school, 91 (36.8%) had completed high school or an equivalent qualification, and 116 (47.0%) were university graduates. The total number of participants in the study was 247, of whom 145 (58.7%) were married, 68 (27.5%) were employed, 53 (21.5%) reported exercising, and 77 (31.2%) had a comorbidity. The mean duration since PMOS diagnosis was 4.0 ± 5.14 years, and the mean age at menarche was 13.0 ± 1.38 years. A family history of PMOS was reported by 91 (36.8%) participants, of whom 67 (73.6%) reported an affected first-degree relative. Infertility treatment was reported by 95 (38.5%) participants, and polycystic ovarian morphology on ultrasonography was present in 244 (98.8%) participants. Participants in the 25–40 age group exhibited significantly higher BMI, waist, hip, neck, and mid-upper arm circumferences, waist-to-hip and waist-to-height ratios, body fat percentage, and lean body mass compared to those in the 19–24 age group (all *p* < 0.05; Table 1). Participants in the 25–40 age group exhibited significantly higher mean PCOSQ-50 total scores in comparison to those in the 19–24 age group (*p* = 0.002). No significant differences were observed between age groups in the six PCOSQ-50 domain scores or in BSQ-34, BDI, CQI, DII, and HEI-2010 scores (all *p*>0.05; Table 1).

**Table 1:** Mean and standard deviation values of individuals' anthropometric measurements, PCOSQ-50, BSQ-34, BDI, CQI, DII and HEI-2010 scores (mean ± SD).

Variables	19–24 years (*n* = 116)	25–40 years (*n* = 131)	Total (*n* = 247)	*t* test	*p–*value
BMI (kg/m^2^)	24.9 ± 5.46	27.7 ± 6.72	26.4 ± 6.31	−3.685	**< 0.001**
Waist circumference (cm)	81.0 ± 13.95	88.4 ± 15.28	84.9 ± 15.08	−3.912	**< 0.001**
Hip circumference (cm)	101.2 ± 12.93	106.5 ± 13.83	104.0 ± 13.65	−3.104	**0.002**
Mid–upper arm circumference (cm)	27.9 ± 3.56	29.7 ± 4.15	28.8 ± 4.98	−3.668	**< 0.001**
Neck circumference (cm)	32.2 ± 3.00	33.9 ± 3.38	33.1 ± 3.31	−4.103	**< 0.001**
Waist/hip ratio	0.8 ± 0.07	0.83 ± 0.07	0.81 ± 0.07	−3.125	**0.002**
Waist/height ratio	0.5 ± 0.08	0.54 ± 0.09	0.52 ± 0.09	−3.957	**< 0.001**
Body fat (%)	29.6 ± 8.25	32.9 ± 8.77	31.3 ± 8.66	−2.944	**0.004**
Lean body mass (kg)	43.4 ± 5.05	45.2 ± 6.04	44.4 ± 5.65	−2.402	**0.017**
PCOSQ−50					
Psychosocial and emotional	36.6 ± 9.13	36.5 ± 11.04	36.5 ± 10.17	0.037	0.970
Fertility	27.0 ± 5.50	26.2 ± 5.32	26.6 ± 5.41	1.210	0.227
Sexual function	34.9 ± 7.80	35.5 ± 7.75	35.3 ± 7.75	−0.472	0.638
Obesity and menstrual disorders	31.2 ± 7.51	30.5 ± 7.39	30.9 ± 7.44	0.761	0.447
Hirsutism	20.0 ± 8.93	20.0 ± 8.59	20.0 ± 8.73	−0.044	0.965
Coping	31.8 ± 3.56	31.5 ± 3.77	31.7 ± 3.66	0.710	0.478
Total	161.0 ± 24.81	171.3 ± 27.34	166.5 ± 26.62	−3.070	**0.002**
BSQ−34	81.2 ± 25.56	85.5 ± 26.09	83.5 ± 25.88	−1.304	0.194
BDI	11.0 ± 5.28	10.2 ± 5.10	10.6 ± 5.18	1.209	0.228
CQI	11.5 ± 3.07	12.1 ± 3.31	11.8 ± 3.21	−1.513	0.132
DII	3.7 ± 1.76	3.4 ± 1.74	3.6 ± 1.75	1.431	0.154
HEI−2010	48.0 ± 14.94	51.7 ± 14.84	50.0 ± 14.96	−1.901	0.059
	**19–24 years** ***n*** **(%)**	**25–40 years** ***n*** **(%)**	**Total** ***n*** **(%)**	**χ2**	***p–*** **value**
BSQ−34					
No body shape concern	66 (56.9)	56 (42.7)	122 (49.4)	6.166	0.104
Mild body shape concern	36 (31.0)	52 (39.7)	88 (35.6)		
Moderate body shape concern	11 (9.5)	21 (16.0)	32 (13.0)		
Severe body shape concern	3 (2.6)	2 (1.5)	5 (2.0)		
BDI					
Minimal depressive symptoms	47 (40.5)	59 (45.0)	106 (42.9)	5.007	0.171
Mild depressive symptoms	58 (50.0)	55 (42.0)	113 (45.7)		
Moderate depressive symptoms	9 (7.8)	17 (13.0)	26 (10.5)		
Severe depressive symptoms	2 (1.7)	–	–		
HEI−2010					
Good	2 (1.7)	4 (3.1)	6 (2.4)	0.880	0.644
Fair	50 (43.1)	61 (46.6)	111 (45.0)		
Bad	64 (55.2)	66 (50.4)	130 (52.6)		

^*t*^ Two independent samples t test. **χ^2^** Chi–square independence test. Significant values are indicated in bold. PCOSQ-50, polycystic ovary syndrome questionnaire-50; BSQ-34, body shape questionnaire-34; BDI, beck depression inventory; CQI, carbohydrate quality index; DII, diet inflammatory index; HEI-2010, healthy eating index-2010.

As demonstrated in Table 2, a number of PCOSQ-50 domain scores exhibited substantial variation across BMI categories. Participants with obesity exhibited lower psychosocial, emotional, and fertility domain scores in comparison to those with normal weight and overweight (*p* < 0.05). Furthermore, a substantial disparity was observed across BMI categories in the domains of obesity and menstrual disorders, with a *p*-value of less than 0.01. The PCOSQ-50 total score was found to be significantly lower in participants with obesity compared to those with a normal weight (*p* = 0.007).

**Table 2:** Mean and standard deviation values of PCOSQ-50, BSQ-34, BDI, CQI, DII and HEI-2010 scores according to individuals' BMI (kg/m^2^) classification (mean ± SD).

Variables	Normal weight (*n* = 128)	Overweight (*n =* 62)	Obese (*n =* 57)	*F*	*p-*value/Tukey
PCOSQ−50					
Psychosocial and emotional	38.6 ± 10.61 ^a^	36.6 ± 8.28 ^b^	31.7 ± 9.47 ^c^	9.960	**< 0.001** (a–c) (b–c)
Fertility	27.2 ± 5.38 ^a^	27.3 ± 5.02 ^b^	24.4 ± 5.41 ^c^	6.134	**0.003** (a–c) (b–c)
Sexual function	36.1 ± 7.70	36.6 ± 6.99	33.0 ± 8.11	3.086	0.049
Obesity and menstrual disorders	34.0 ± 7.77 ^a^	29.0 ± 5.06 ^b^	25.9 ± 5.10 ^c^	33.315	**< 0.001** (a–b) (b–c) (a–c)
Hirsutism	21.0 ± 8.54	19.3 ± 8.85	18.6 ± 8.93	1.694	0.186
Coping	32.5 ± 3.63 ^a^	31.1 ± 3.63 ^b^	30.4 ± 3.38 ^c^	7.489	**0.001** (a–b) (a–c)
Total	170.2 ± 28.26 ^a^	167.6 ± 23.40^b^	157.0 ± 24.12^c^	5.077	**0.007** (a–c)
BSQ−34	68.5 ± 21.36 ^a^	88.3 ± 15.48 ^b^	111.8 ± 17.07 ^c^	104.189	**< 0.001** (a-b) (b-c) (a-c)
BDI	9.1 ± 5.53 ^a^	11.5 ± 4.24 ^b^	12.8 ± 4.27 ^c^	12.525	**< 0.001** (a–b) (a–c)
CQI	11.9 ± 3.18	11.7 ± 3.18	11.6 ± 3.35	0.194	0.824
DII	3.5 ± 1.82	3.4 ± 1.70	3.9 ± 1.65	1.613	0.201
HEI−2010	51.1 ± 15.44	48.9 ± 13.66	48.5 ± 15.27	0.792	0.454
	**Normal weight (*****n*** **=** **128)**	**Overweight (*****n** **=*** **62)**	**Obese (*****n** **=*** **57)**	**χ2**	***p–*** **value**
BSQ−34					
No body–shape concern	101 (78.9)	20 (32.3)	1 (1.8)	134.976	**< 0.001**
Mild body–shape concern	23 (18.0)	37 (59.7)	28 (49.1)		
Moderate–to–severe body–shape concern	4 (3.1)	5 (8.0)	28 (49.1)		
BDI					
Minimal depressive symptoms	73 (57.0)	20 (32.3)	13 (22.8)	29.683	**< 0.001**
Mild depressive symptoms	48 (37.5)	35 (56.5)	30 (52.6)		
Moderate–severe depressive symptoms	7 (5.5)	7 (11.2)	14 (24.6)		
HEI−2010					
Good–fair	67 (52.3)	24 (38.7)	26 (45.6)	3.206	0.201
Bad	61 (47.7)	38 (61.3)	31 (54.4)		

^*a, b, c*^ Different lowercase superscript letters indicate significant pairwise differences according to Tukey's post-hoc test; groups sharing the same letter do not differ significantly. Significant values are indicated in bold. F:One-Way ANOVA test. **χ^2^** Chi-square independence test. BMI, body mass index; PCOSQ-50, polycystic ovary syndrome questionnaire-50; BSQ-34, body shape questionnaire-34; BDI, beck depression inventory; CQI, carbohydrate quality index; DII, diet inflammatory index; HEI-2010, healthy eating index-2010.

Furthermore, a significant divergence in BSQ-34 and BDI scores was observed across BMI categories, with higher scores recorded in participants with obesity compared to those with a normal BMI (*p* < 0.001). No significant differences were observed in CQI, DII, or HEI-2010 scores across BMI categories (all *p*>0.05; Table 2).

The PCOSQ-50 total and several domain scores exhibited significant variation according to depressive symptom severity (see Table 3). Participants exhibiting minimal depressive symptoms demonstrated higher psychosocial and emotional, obesity and menstrual disorders, hirsutism, coping, and total PCOSQ-50 scores in comparison to those manifesting mild or moderate-to-severe depressive symptoms (all *p* < 0.001). The sexual function domain also differed significantly across depressive symptom categories (*p* = 0.020), whereas no significant difference was observed in the fertility domain (*p* = 0.082).

**Table 3:** Mean and standard deviation values of PCOSQ-50 subscales and total scores of individuals according to BDI classification (mean ± SD).

PCOSQ-50 domains	Beck depression inventory			F	*p–*value
	Minimal depressive symptoms (*n* = 106)	Mild depressive symptoms (*n* = 113)	Moderate–to–severe depressive symptoms (*n* = 28)		
Psychosocial and emotional	39.6 ± 10.14 ^a^	35.1 ± 9.51 ^b^	30.7 ± 9.34 ^b^	11.322	**< 0.001**
Fertility	27.4 ± 5.47	26.2 ± 5.20	25.1 ± 5.73	2.525	0.082
Sexual function	37.3 ± 7.33 ^a^	34.5 ± 7.41 ^a−*b*^	32.3 ± 8.81 ^b^	4.027	**0.020**
Obesity and menstrual disorders	33.4 ± 7.36 ^a^	29.5 ± 6.79 ^b^	26.9 ± 7.32 ^b^	13.052	**< 0.001**
Hirsutism	22.8 ± 8.01 ^a^	17.8 ± 8.93 ^b^	18.4 ± 7.86 ^b^	10.027	**< 0.001**
Coping	32.5 ± 3.31 ^a^	31.5 ± 3.29 ^a^	29.1 ± 4.98 ^b^	11.064	**< 0.001**
Total	175.7 ± 27.61 ^a^	160.8 ± 23.20 ^b^	154.4 ± 25.32 ^b^	12.949	**< 0.001**

^*a, b*^ Different lowercase superscript letters indicate significant pairwise differences according to Tukey's post–hoc test; groups sharing the same letter do not differ significantly.

F:One–Way ANOVA test. Significant values are indicated in bold. PCOSQ−50, polycystic ovary syndrome quality of life questionnaire−50; BDI, beck depression inventory.

PCOSQ-50 total and several domain scores exhibited significant variation across BSQ-34 body shape concern categories (see Table 4). Participants with moderate-to-severe body shape concern exhibited lower psychosocial and emotional, obesity and menstrual disorders, coping, and total PCOSQ-50 scores in comparison to participants with lower levels of body shape concern (all *p* < 0.001). No significant differences were observed in the fertility, sexual function, or hirsutism domains across BSQ-34 categories (all *p*>0.05).

**Table 4:** Mean and standard deviation values of PCOSQ−50 subscale and total scores of individuals according to BSQ−34 classification (mean ± SD).

PCOSQ-50 domains	Body shape questionnaire−34			*F*	*p–*value
	No body–shape concern (*n* = 122)	Mild body–shape concern (*n* = 88)	Moderate–to–severe body–shape concern (*n*=37)		
Psychosocial and emotional	39.2 ± 10.15 ^a^	35.8 ± 9.08 ^b^	29.6 ± 9.31 ^c^	14.312	**< 0.001**
Fertility	27.2 ± 5.24	26.4 ± 5.37	24.8 ± 5.75	3.005	0.051
Sexual function	36.3 ± 7.53	35.6 ± 6.83	32.2 ± 9.44	2.782	0.065
Obesity and menstrual disorders	34.6 ± 7.07 ^a^	28.3 ± 5.68 ^b^	24.5 ± 5.10 ^c^	47.592	**< 0.001**
Hirsutism	20.4 ± 8.54	20.1 ± 8.86	18.4 ± 9.13	0.769	0.465
Coping	32.5 ± 3.39 ^a^	31.5 ± 3.32 ^a^	29.3 ± 4.32 ^b^	11.756	**< 0.001**
Total	172.1 ± 26.86 ^a^	165.5 ± 23.29 ^a^	150.1 ± 26.86 ^b^	10.564	**< 0.001**

^*a, b, c*^ Different lowercase superscript letters indicate significant pairwise differences according to Tukey's post-hoc test; groups sharing the same letter do not differ significantly.

F:One–Way ANOVA test. Significant values are indicated in bold. PCOSQ−50, polycystic ovary syndrome quality of life questionnaire−50; BSQ−34, body shape questionnaire−34.

The results of the correlation analyses demonstrated a negative correlation between PCOSQ-50 total scores and BSQ-34 (*r* = −0.342, *p* < 0.001), BDI (*r* = −0.393, *p* < 0.001), and BMI (*r* = −0.198, *p* = 0.002). The results of the study demonstrated a positive correlation between BSQ-34 and BDI (*r* = 0.551, *p* < 0.001) and BMI (*r* = 0.705, *p* < 0.001). In addition, a positive correlation was identified between BDI and BMI (*r* = 0.393, *p* < 0.001) (see Figure 1). Among the dietary indices, DII exhibited a negative correlation with CQI and HEI-2010, while CQI demonstrated a positive correlation with HEI-2010 (all *p* < 0.001).

**Figure 1:**
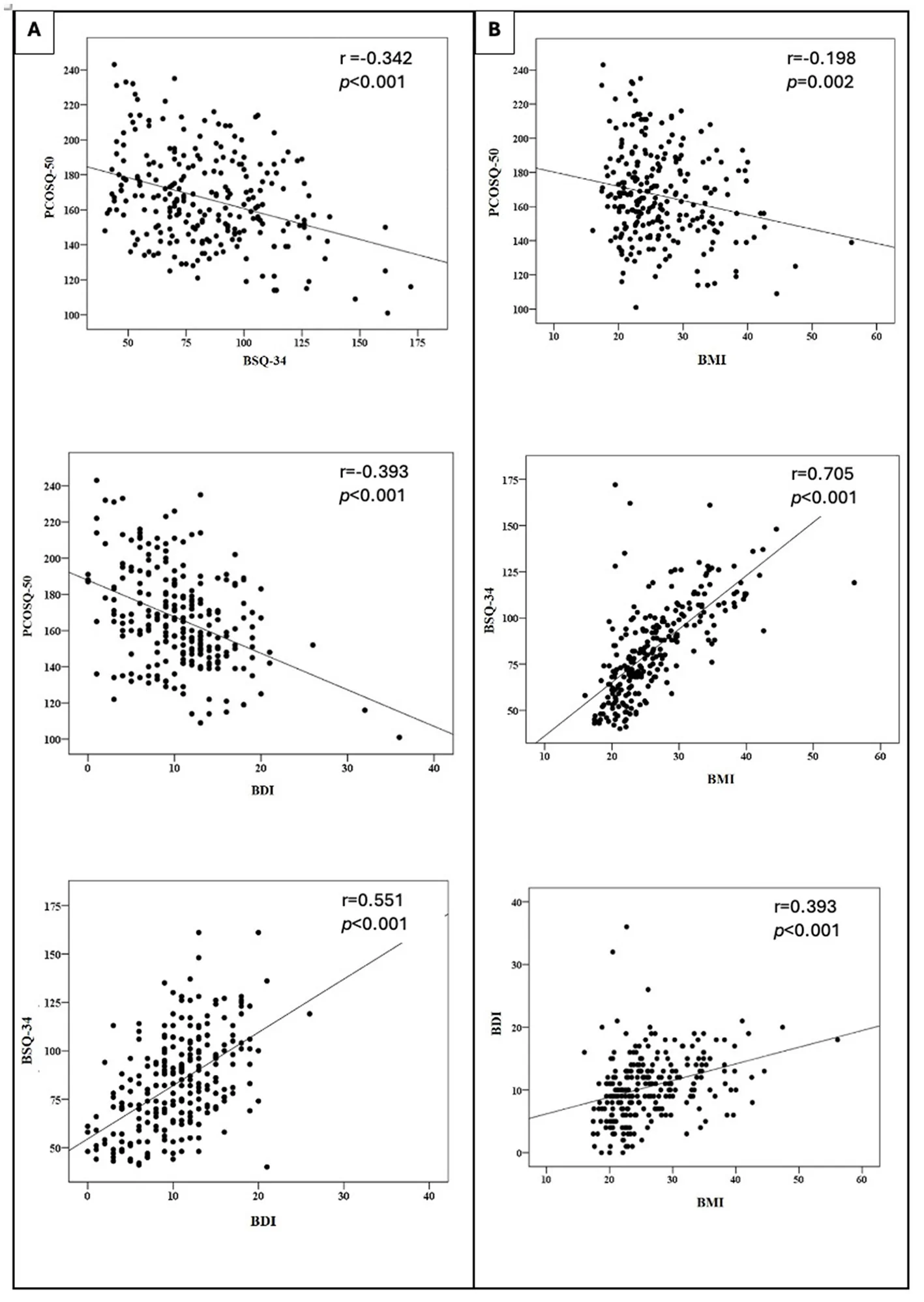
Correlation between scales **(A)**, correlation between BMI and scales **(B)**.

The regression assumptions were checked before interpreting the models. No important multicollinearity was observed, with tolerance values ranging from 0.553 to 0.959 and VIF values from 1.043 to 1.808. Inspection of the residual plots did not indicate a clear problem with linearity or homoscedasticity, and the P–P plots suggested that the residuals were approximately normally distributed. A few cases had standardized residuals above ±3 in the BSQ-34 and BDI models. However, the maximum Cook's distance values were 0.157 and 0.141, respectively, and the maximum leverage value was 0.138, suggesting that these cases did not have a substantial influence on the models. The maximum Cook's distance was 0.037 for the PCOSQ-50 model. Durbin–Watson values were 1.889 for PCOSQ-50, 1.602 for BSQ-34, and 1.701 for BDI.

In the multiple linear regression analyses, age group, marital status, BMI, and duration since PMOS diagnosis were found to be independently associated with PCOSQ-50 total scores (adjusted R^2^ = 0.38, *p* < 0.001). In comparison with participants within the 19–24 age range, those within the 25–40 age range exhibited elevated PCOSQ-50 total scores (B = 7.089, 95% CI: 1.161–13.018, *p* = 0.019). Furthermore, marital status emerged as a significant factor, with married participants demonstrating higher scores in comparison to their single counterparts (B = 30.186, 95% CI: 24.378–35.994, *p* < 0.001). A higher BMI (B = −1.418, 95% CI: −1.866 to −0.969, *p* < 0.001) and a longer duration since PMOS diagnosis (B = −0.798, 95% CI: −1.341 to −0.254, *p* = 0.004) were found to be associated with lower PCOSQ-50 total scores.

For BSQ-34, comorbidity and BMI were found to be independently associated with scores (adjusted R^2^ = 0.50, *p* < 0.001). Participants with comorbidity demonstrated lower BSQ-34 scores in comparison to those without comorbidity (B = −5.537, 95% CI: −10.580 to −0.494, *p* = 0.032). Conversely, higher BMI was associated with higher BSQ-34 scores (B = 2.869, 95% CI: 2.478–3.260, *p* < 0.001). In the case of BDI, it was demonstrated that age group and BMI were independently associated with scores (adjusted R^2^ = 0.11, *p* < 0.001). In the study, participants in the 25–40 age group demonstrated lower BDI scores in comparison to those in the 19–24 age group (B = −1.903, 95% CI: −3.284 to −0.521, *p* = 0.007). Additionally, higher BMI was associated with higher BDI scores (B = 0.284, 95% CI: 0.180–0.389, *p* < 0.001) (Table 5).

**Table 5:** Multiple linear regression analyses of factors associated with PCOSQ−50, BSQ−34, and BDI scores.

Variables	B	SE	β	*p–*value	%95 CI	Tolerance	VIF
PCOSQ−50 total score^a^							
Age group (ref: 19–24 years)	7.089	3.009	0.133	**0.019**	(1.161; 13.018)	0.787	1.271
Marital status (ref: single)	30.186	2.948	0.559	**< 0.001**	(24.378; 35.994)	0.843	1.187
Exercise (ref: non–exercise)	−0.878	3.384	−0.014	0.795	(-7.544; 5.787)	0.920	1.087
Comorbidity (ref: none)	4.152	2.937	0.072	0.159	(-1.634; 9.939)	0.959	1.043
BMI	−1.418	0.228	−0.336	**< 0.001**	(-1.866;−0.969)	0.862	1.160
PMOS diagnosis time (years)	−0.798	0.276	−0.154	**0.004**	(-1.341;−0.254)	0.888	1.126
CQI	−0.535	0.530	−0.064	0.314	(-1.579; 0.509)	0.616	1.624
DII	−0.399	0.950	−0.026	0.675	(-2.269; 1.472)	0.642	1.558
HEI−2010	0.053	0.120	0.030	0.661	(-0.184; 0.289)	0.553	1.808
BSQ−34 score^b^							
Age group (ref: 19–24 years)	−4.628	2.623	−0.089	0.079	(-9.794; 0.539)	0.787	1.271
Marital status (ref: single)	1.009	2.569	0.019	0.695	(-4.052; 6.071)	0.843	1.187
Exercise (ref: non-exercise)	5.661	2.949	0.090	0.056	(-0.149; 11.470)	0.920	1.087
Comorbidity (ref: none)	−5.537	2.560	−0.099	**0.032**	(-10.580;−0.494)	0.959	1.043
BMI	2.869	0.198	0.700	**< 0.001**	(2.478; 3.260)	0.862	1.160
PMOS diagnosis time (years)	0.053	0.240	0.010	0.826	(-0.421; 0.526)	0.888	1.126
CQI	−0.513	0.462	−0.064	0.268	(-1.422; 0.397)	0.616	1.624
DII	−0.713	0.828	−0.048	0.390	(-2.344; 0.917)	0.642	1.558
HEI−2010	−0.043	0.105	−0.025	0.681	(-0.249; 0.163)	0.553	1.808
BDI score^c^							
Age group (ref: 19–24 years)	−1.903	0.701	−0.183	**0.007**	(-3.284;−0.521)	0.787	1.271
Marital status (ref: single)	0.018	0.687	0.002	0.979	(-1.336; 1.371)	0.843	1.187
Exercise (ref: non–exercise)	0.886	0.789	0.070	0.262	(-0.667; 2.440)	0.920	1.087
Comorbidity (ref: none)	−0.482	0.685	−0.043	0.482	(-1.830; 0.867)	0.959	1.043
BMI	0.284	0.053	0.346	**< 0.001**	(0.180; 0.389)	0.862	1.160
PMOS diagnosis time (years)	0.078	0.064	0.077	0.228	(-0.049; 0.204)	0.888	1.126
CQI	−0.079	0.124	−0.049	0.521	(-0.323; 0.164)	0.616	1.624
DII	−0.324	0.221	−0.110	0.144	(-0.760; 0.112)	0.642	1.558
HEI−2010	−0.025	0.028	−0.072	0.375	(-0.080; 0.030)	0.553	1.808

^a^F = 17.853, p < 0.001; adjusted R^2^ = 0.38. ^b^F = 28.649, p < 0.001; adjusted R^2^ = 0.50.

^c^F = 4.526, p < 0.001; adjusted R^2^ = 0.11.

B, unstandardized coefficient; β, standardized coefficient; Ref, reference; SE, standard error; CI, confidince interval; adjusted R^2^, adjusted coefficient of determination; VIF, variance inflation factor; BMI, body mass index; PMOS, polyendocrine metabolic ovarian syndrome; PCOSQ-50, polycystic ovary syndrome questionnaire-50; BSQ-34, body shape questionnaire-34; BDI, beck depression inventory; CQI, carbohydrate quality index; DII, diet inflammatory index; HEI-2010, healthy eating index-2010. Significant values are indicated in bold.

## 4 Discussion

Poliendocrin metabolic ovarian syndrome has been linked to a variety of metabolic, reproductive and psychological challenges, which have the potential to compromise psychosocial wellbeing and disease-specific quality of life. A substantial body of research has repeatedly indicated the presence of elevated depressive symptoms, psychological distress, eating-related issues, and body-image concerns among women diagnosed with PMOS (26**?** –29). In line with the findings of the present study, previous research has demonstrated an association between PMOS-specific quality of life, depressive symptoms, body shape concerns, and BMI. Rather than identifying each of these associations as individually novel, an important contribution of the present study is the concurrent evaluation of PMOS-specific quality of life, depressive symptoms, body shape concerns, anthropometric characteristics, and multiple dimensions of dietary quality within the same sample.

Anthropometric status has been demonstrated to be closely related to the clinical and psychosocial burden of PMOS. A plethora of studies have previously reported an association between excess body weight, central adiposity, and the clinical manifestations of PMOS (30–33). In the present study, participants aged 25–40 years exhibited higher mean BMI and several anthropometric measures in comparison to those aged 19–24 years. In consideration of the cross-sectional design, these findings do not demonstrate an increase in obesity with age, but rather indicate that the older age group in this sample exhibited higher anthropometric measurements. Obesity and PMOS have been demonstrated to be closely interrelated, with insulin resistance, hyperinsulinemia, androgen excess, and adipose-tissue dysfunction among the mechanisms proposed to link excess adiposity with the metabolic and reproductive manifestations of PMOS (34, 35). Conversely, PMOS-related metabolic and psychological features have also been demonstrated to complicate weight management, thus suggesting a complex and potentially bidirectional relationship rather than a simple cause-and-effect pathway (34). However, the directionality of these relationships cannot be determined from the present cross-sectional study.

PMOS-related clinical manifestations have been demonstrated to be associated with a poorer disease-specific quality of life, particularly in psychosocial and symptom-related domains (36–38). In the present study, a negative correlation was identified between BMI and the PCOSQ-50 total score. Participants with obesity exhibited lower PCOSQ-50 total scores in comparison to those with a normal BMI. A number of differences were also observed in several domains of the PCOSQ-50, including the psychosocial and emotional status, fertility, obesity and menstrual disorders, and coping. In addition, a higher BMI remained independently associated with lower PCOSQ-50 total scores in the multivariable model. Recent studies have yielded equivocal results regarding the relationship between BMI and PMOS-specific quality of life (30, 37, 38). Such inconsistencies may be indicative of variations in population characteristics, symptom burden, weight status, and methodological approaches. The findings of this study provide evidence to support the hypothesis that there is an association between higher BMI and poorer PMOS-specific quality of life. However, the study does not provide sufficient evidence to establish a causal relationship.

Another salient dimension of the psychosocial burden of PMOS is represented by body-image concern. The concept of body image is understood to encompass perceptions, attitudes, and feelings related to physical appearance and functioning (39). Research has repeatedly indicated that women diagnosed with PMOS frequently report dissatisfaction with their body image (40**?** –42). In the present study, BSQ-34 scores were found to be positively correlated with both BMI and BDI scores. Furthermore, participants who exhibited moderate-to-severe body shape concern demonstrated lower PCOSQ-50 total scores and lower scores in several PCOSQ-50 domains. In the multivariable analysis, BMI demonstrated a positive and independent association with BSQ-34 scores. The findings of this study indicate a close association between body shape concern and both anthropometric status and psychosocial wellbeing in women diagnosed with PMOS. In light of the acknowledged psychological distress associated with PMOS, contemporary global guidelines underscore the necessity of incorporating emotional wellbeing, quality of life, and weight stigma into comprehensive care interventions (43).

Depressive symptoms represent a significant component of the psychosocial burden associated with PMOS. As demonstrated in previous studies, this population has exhibited elevated levels of depressive and anxiety symptoms, with concomitant associations observed among depression, obesity, body-image disturbance, and diminished quality of life (26, 27, 36, 42, 44–47). Current international evidence-based recommendations underscore the high prevalence of psychological features in PMOS and emphasize their recognition as part of clinical assessment and management (43). In the present study, BDI scores were found to be positively correlated with BMI and BSQ-34 scores, and negatively correlated with PCOSQ-50 total scores. Participants exhibiting minimal depressive symptoms demonstrated higher total scores on the PCOSQ-50 and across several domains when compared to those experiencing more severe depressive symptoms. Furthermore, a higher BMI was found to be independently associated with higher BDI scores in the multivariable model. The findings of this study lend support to the hypothesis of coexistence and interrelationship among depressive symptoms, body shape concerns, BMI, and PMOS-specific quality of life, rather than demonstrating a causal pathway among these factors. Furthermore, they emphasize the necessity of incorporating psychological wellbeing into the management of PMOS, emphasizing its significance alongside metabolic and reproductive features (43).

The quality of one's diet has been identified as a further potentially pertinent component in the management of PMOS. A substantial body of research has previously employed the Healthy Eating Index as a tool to assess diet quality in women diagnosed with PMOS. The findings of these studies have consistently indicated that the diets of these women do not meet the recommended standards of nutritional adequacy (48–51). In the present study, the mean HEI-2010 score was found to be within the poor diet-quality range, with more than half of the participants being classified as having poor diet quality. However, no significant differences in HEI-2010 scores were observed across the BMI categories. This finding is in contrast to the results reported by Rodrigues et al., who found an association between a poorer diet quality and overweight/obesity among women with PMOS (51). Such disparities may be indicative of variations in dietary assessment methodologies, population characteristics, and anthropometric profiles. Consequently, despite the prevalence of suboptimal diet quality in the present sample, no significant correlation was observed between diet quality and BMI category. However, current international recommendations emphasize that a healthy diet and regular physical activity are integral components of lifestyle management in PMOS, with benefits extending beyond weight management alone (43).

The CQI and DII provide complementary perspectives on dietary quality by reflecting carbohydrate quality and the inflammatory potential of the diet, respectively. A number of earlier studies have indicated an association between higher CQI and lower general or abdominal obesity, as well as more favorable metabolic characteristics. However, the findings have not been uniform across different populations (52–54). In the present study, CQI did not differ significantly across BMI categories; however, CQI was positively correlated with HEI-2010 and negatively correlated with DII. The conceptual consistency of these relationships is attributable to the fact that diets characterized by higher-quality carbohydrate sources, including whole grains, fruits, vegetables, and dietary fiber, may simultaneously contribute to better overall diet quality and a less pro-inflammatory dietary profile.

Greater dietary inflammatory potential has also been investigated in relation to PMOS, adiposity, and metabolic health (46, 55, 56). In the present study, the mean DII score was positive, indicating a predominantly pro-inflammatory dietary profile. However, DII did not differ significantly across BMI categories, and CQI, DII, and HEI-2010 were not independently associated with PCOSQ-50, BSQ-34, or BDI scores in the multivariable models. The findings of this study indicate that, within the context of the present sample, the psychosocial outcomes under scrutiny demonstrated a stronger correlation with anthropometric and demographic or clinical characteristics in comparison to the dietary indices that were the subject of evaluation. Nevertheless, dietary interventions and weight management have been investigated for their potential to improve inflammatory and metabolic characteristics in women with PMOS, supporting the continued relevance of dietary assessment within comprehensive management (35, 43). As the dietary intake and outcomes examined in the present study were assessed cross-sectionally, the absence of independent associations should not be interpreted as evidence that dietary quality is unimportant in PMOS.

The current findings demonstrate a closely interconnected pattern involving PMOS-specific quality of life, depressive symptoms, body shape concerns, and BMI, whereas the dietary indices showed weaker associations with these psychosocial outcomes. By concurrently examining disease-specific quality of life, psychological status, body shape concern, anthropometric characteristics, and several complementary dietary indices, this study provides an integrated perspective on factors associated with the health and wellbeing of women with PMOS. The findings support a multidimensional approach to PMOS assessment that considers psychological and body-image concerns alongside anthropometric and nutritional characteristics. However, given the cross-sectional design, the observed relationships should be interpreted as associations rather than causal effects.

## 5 Strengths and limitations

A strength of the present study is the concurrent evaluation of PMOS-specific quality of life, depressive symptoms, body shape concerns, anthropometric characteristics, and multiple dimensions of dietary quality within the same sample, providing an integrated assessment of factors associated with the multidimensional burden of PMOS. Nevertheless, several limitations should be considered when interpreting the findings. First, the cross-sectional design precludes conclusions regarding causality or the direction of the observed associations. Second, the study was conducted at a single center and did not include a control group, which may limit the generalizability of the findings. In addition, women with a diagnosed psychiatric disorder requiring current pharmacological treatment were excluded. This criterion may have resulted in underrepresentation of women with more severe psychological morbidity and should therefore be considered when interpreting the findings related to depressive symptoms and quality of life. Dietary intake was assessed using a single 24-h dietary recall, which may not adequately reflect habitual dietary intake and may be subject to day-to-day variation and recall bias. Consequently, the HEI-2010, CQI, and DII estimates should be interpreted with appropriate caution. Finally, because multiple comparisons were performed using the PCOSQ-50 total and domain scores without adjustment for multiple testing, the possibility of an increased Type I error should be considered when interpreting these findings.

## 6 Conclusion

The present study underscores the interrelationships among PMOS-specific quality of life, depressive symptoms, body shape concerns, and anthropometric characteristics in women diagnosed with PMOS. A higher BMI was found to be associated with lower PCOSQ-50 total scores and greater body shape concerns and depressive symptoms. In addition, greater depressive symptoms and body shape concerns were also found to be associated with a poorer PMOS-specific quality of life. Despite the observation of substandard diet quality and a predominantly pro-inflammatory dietary profile within the study population, the dietary indices demonstrated comparatively weaker associations with the psychosocial outcomes examined. The present study provides an integrated perspective on the multidimensional burden of PMOS by concurrently evaluating psychological, anthropometric, quality-of-life, and dietary characteristics. These findings lend support to comprehensive management approaches that consider psychological wellbeing and body-image concerns alongside anthropometric and nutritional characteristics. However, given the cross-sectional design and sample characteristics, it is not possible to establish causal relationships, and the generalizability of the findings should be considered with caution. Further prospective studies are required to elucidate the direction and clinical relevance of these associations.

## Data Availability

The datasets presented in this article are not readily available because the datasets are not publicly available due to ethical and privacy restrictions related to human participant data. Requests to access the datasets should be directed to Esra Irmak, irmakkess@gmail.com.
